# A randomised controlled trial and cost effectiveness study of systematic screening (targeted and total population screening) versus routine practice for the detection of atrial fibrillation in the over 65s: (SAFE) [ISRCTN19633732]

**DOI:** 10.1186/1471-2261-4-12

**Published:** 2004-07-29

**Authors:** Dawn Swancutt, Richard Hobbs, David Fitzmaurice, Jonathan Mant, Ellen Murray, Sue Jowett, James Raftery, Stirling Bryan, Michael Davies, Gregory Lip

**Affiliations:** 1 Department of Primary Care and General Practice, The University of Birmingham, Birmingham, UK; 2 Health Economics Facility, The University of Birmingham, Birmingham, UK; 3 Department of Cardiology, Selly Oak Hospital, Birmingham, UK; 4 University Department of Medicine, City Hospital, Birmingham, UK

## Abstract

**Background:**

Atrial fibrillation (AF) has been recognised as an important independent risk factor for thromboembolic disease, particularly stroke for which it provides a five-fold increase in risk. This study aimed to determine the baseline prevalence and the incidence of AF based on a variety of screening strategies and in doing so to evaluate the incremental cost-effectiveness of different screening strategies, including targeted or whole population screening, compared with routine clinical practice, for detection of AF in people aged 65 and over. The value of clinical assessment and echocardiography as additional methods of risk stratification for thromboembolic disease in patients with AF were also evaluated.

**Methods:**

The study design was a multi-centre randomised controlled trial with a study population of patients aged 65 and over from 50 General Practices in the West Midlands. These purposefully selected general practices were randomly allocated to 25 intervention practices and 25 control practices. GPs and practice nurses within the intervention practices received education on the importance of AF detection and ECG interpretation. Patients in the intervention practices were randomly allocated to systematic (n = 5000) or opportunistic screening (n = 5000). Prospective identification of pre-existing risk factors for AF within the screened population enabled comparison between high risk targeted screening and total population screening. AF detection rates in systematically screened and opportunistically screened populations in the intervention practices were compared to AF detection rate in 5,000 patients in the control practices.

## Background

Atrial fibrillation (AF) has been recognised as an important independent risk factor for thromboembolic disease, particularly stroke with which it is associated with a five fold increase in risk [1]. There are few data on the prevalence of AF in the United Kingdom. Local data derived from the Echocardiographic Heart of England Screening (ECHOES) study suggested a prevalence of AF in people over the age of 65 of 3.8% (95% CI: 2.5–5.1) [2]. A review of four large community based studies of AF suggested that the overall community prevalence in the United States is 0.89% [3]. In these studies, the prevalence increased sharply with age: 2.3% of people aged 40 or over; 5.9% of people aged over 65 (higher than the local estimate), and 10% of those over 80. The vast majority (84%) of people with AF are over the age of 65. AF is a particularly important risk factor for stroke in the elderly – while 15% of all strokes are associated with the arrhythmia, it is associated with 36% of strokes in people over the age of 80. The incidence of new cases of AF in people over the age of 65 is of the order of 1% per annum [4].

Screening for AF in the elderly fulfils many of the Wilson-Jungner criteria for a screening programme [5]. It is a common and important condition which can be diagnosed by means of a simple test, and the risk of serious sequelae such as stroke can be dramatically reduced by treatment.

One UK study has compared systematic nurse-led screening with prompted opportunistic case finding for AF in primary care [6]. This small scale study (four practices, n = 3001) demonstrated that systematic nurse-led screening detected more cases than opportunistic case finding, however most of those cases detected were already diagnosed. Two further single practice based studies have investigated the role of practice nurses in the screening process [7], and whole population screening [8]. 5% of total NHS expenditure can be attributed to stroke, and there would be expected to be about 1,000 new cases of stroke per annum in a typical health authority of a half million population. Therefore, any programme that might lead to an important reduction in stroke incidence needs serious consideration, both because of the potential for health gain, and the potential for reduced overall NHS expenditure. Screening for AF might be one such programme since, in population terms, AF is an important risk factor for stroke and anticoagulation provides a highly effective treatment to reduce this risk. A meta-analysis of randomised controlled trials has shown a 68% relative risk reduction in patients' with AF receiving oral anticoagulation [9]. It has been estimated that optimal treatment of AF in the population might reduce the overall incidence of stroke by 10%. However, before implementing screening programmes, unresolved questions over how the screening should be conducted must be answered.

### The appropriate screening strategy to be employed

#### Opportunistic screening

The simplest strategy was *opportunistic case finding*, where a health care professional took the opportunity to feel a patient's pulse during a consultation. If the pulse is irregular, they might make a clinical diagnosis of AF, or request/perform an electrocardiogram (ECG) as a confirmatory test. However, opportunistic case finding is likely to miss a significant proportion of people who would otherwise have benefited from treatment. For example, detection of hypertension in general practice was traditionally detected in an opportunistic way until the introduction of health checks with the 1990 GP contract. The Health Survey for England shows that in 1991, 42% of the population over the age of 75 had hypertension for which they were not taking any medication [10]. This figure had fallen to 31% by 1994, after the GP contract had taken effect.

#### Targeted screening

One possible approach was to screen patients who are at higher risk of AF – a *targeted screening programme*. Cardiac failure, hypertension and rheumatic heart disease are important precursors of AF [7]. AF is more common in people with a history of myocardial infarction, angina, diabetes mellitus, hyperthyroidism, stroke or transient ischaemic attack (than in people without these conditions) [11]. Most general practices were computerised, and some have disease registers. A targeted screening programme could exploit these to identify such high risk patients, either through disease registers, or through prescribing information on the computerised records.

#### Whole population screening

Another approach was to screen everyone 65 and over (65+) for AF – a *whole population screening programme*.

A modelling exercise using decision analysis to inform on the methodology for this study indicated that there were not sufficient primary data available to recommend which of these (targeted or whole population) would be the optimum policy

#### The most appropriate screening test for AF

12-lead ECG is recognised as the gold standard test, but this test is time consuming (taking at least 15 minutes to perform in an outpatient setting). Therefore, it is important to consider simpler tests. This study assessed simpler methods compared to the gold standard, both in terms of accuracy, time taken and patient acceptability. These include taking the pulse, and simpler ECGs.

#### Interpreting the ECG

Cardiologists offer the most accurate readings of ECGs, but can satisfactory interpretations be obtained by the GP, the practice nurse, or computerised diagnostic software? This study assessed the accuracy of these different approaches to interpreting the ECG.

#### The value of echocardiography

The main treatment options to reduce risk of stroke in patients' with AF are currently warfarin or aspirin. Aspirin is much less effective than warfarin – it achieves a barely significant 21% reduction in stroke risk [9]. However, it is safer to use, since it confers a lower risk of serious haemorrhage. Therefore, in practice, the clinical decision as to which treatment to use depends upon the balance of risks and benefits for the individual patient. Thromboembolic risk is currently determined primarily on clinical criteria. Data from the SPAF study [12] suggested that echocardiography (Echo) may inform on risk stratification, assisting in therapeutic decision making. The role of routine Echo for patients with AF identified in the community remains to be proven. Data also needs to be quantified regarding the cost effectiveness of Echo versus clinical impression alone. Studies have suggested that the clinical utility in people aged over 74 is poor [13,14]. Therefore this study focused on patients aged 65–74. Once somebody has been identified as having AF, should they also receive an echocardiogram to assess their risk of stroke, or is clinical assessment of risk adequate?

#### Optimum strategy

This study, by providing answers to these questions, allowed the optimum strategy for introducing a screening programme for AF in the over 65s to be determined. However, before a decision is made as to whether to institute a screening programme, not only must the question of the best strategy be considered, but also, the question of whether any screening programme at all should be introduced. This study provided data to assist in answering this fundamental question by providing:

i) An accurate estimate of the community prevalence and incidence of AF in over 65s;

ii) An assessment of the health economic implications of screening for AF;

iii) An assessment of the service provision implications of implementing such a programme;

iv) An assessment of the impact on patient quality of life and anxiety after various screening methodologies.

#### Health economics of screening

Although the cost effectiveness of different approaches to screening is often put in terms of the average cost per case detected, such an approach ignores the sensitivity and specificity of the screening test. This is because average cost per case detected focuses entirely on true positives, paying no attention to false positives, false negatives and true negatives. False positives and false negatives impose costs on patients and health services which would be neglected if the focus was confined to true positives [15]. An undue emphasis on the average cost per case detected could justify opportunistic screening of a small number of high risk patients who present, with no consideration of the number of cases missed.

This study compared the incremental cost per case detected for different methods of AF screening. This refers not to the average cost but rather approximates the incremental cost per case detected in moving from one of the screening options to another. Use of incremental cost per case detected by option shows how the cost per additional case detected is likely to increase as the intensity of screening increases. This method has been used to deal with similar uncertainties about the cost effectiveness of screening for other diseases, including breast and colorectal cancer and has been recommended by the US guidelines [16].

### Objectives

#### Primary objective

• To determine baseline prevalence and the incidence of AF based on a variety of screening strategies and in doing so to evaluate the incremental cost-effectiveness, in terms of cost per case identified, of the different screening strategies (targeted or whole population screening) compared with routine clinical practice for detection of AF in people aged 65 and over.

#### Secondary objectives

• To evaluate the relative cost-effectiveness of screening methods for AF diagnosis, comparing 12 lead ECG (gold standard) with pulse taking, lead II rhythm strip from standard ECG limb leads alone and single lead thoracic placement ECG.

• To evaluate the most cost-effective method of test interpretation, comparing cardiologist (gold standard), with GP, practice nurse, or computerised diagnostic software.

• To assess the differing combinations of screening strategies and procedures in terms of patient acceptability and impact on patient quality of life, including any psychological effects of screening.

• To determine the community prevalence of AF in people 65+.

• To evaluate the value of clinical assessment and echocardiography as additional methods of risk stratification for thromboembolic disease in patients with AF.

• To evaluate the service provision implications should screening for AF become a national programme, and identify the optimum screening algorithm for identification of patients with AF.

### Outcome measures

#### Primary outcome

• The incidence of AF according to a variety of screening strategies

• The associated costs providing an incremental cost per case detected. The cost data was collected from an NHS and patient perspective. It has focused on resources required to establish screening, time taken to complete screening and the cost of the equipment.

#### Secondary outcomes

• Cost effectiveness of 4 different methods of screening for AF. The cost data focused on the difference in the cost of the equipment and the time taken for each of the different methods of screening to be completed. This was from both an NHS and patient perspective.

• Cost effectiveness of 4 different methods of ECG interpretation. The cost data focused on the difference in the cost of the grade of staff interpreting the ECG and the accuracy of their interpretation.

• Overall community prevalence and incidence of AF

• Patient acceptability to AF screening was measured using an adapted version of the screening specific questionnaire used in the Colorectal Screening Programme [18]. Patient uptake of screening was also monitored. The impact on quality of life was assessed using EQ-5D [24,25]. Patient anxiety was measured using the Spielberger 6 item Anxiety Questionnaire [17].

• Modelling techniques were used to identify the implications of AF screening on health service provision nationally. This included the effect on echocardiography and anti-coagulation clinic provision.

## Methods

**Figure 1:**
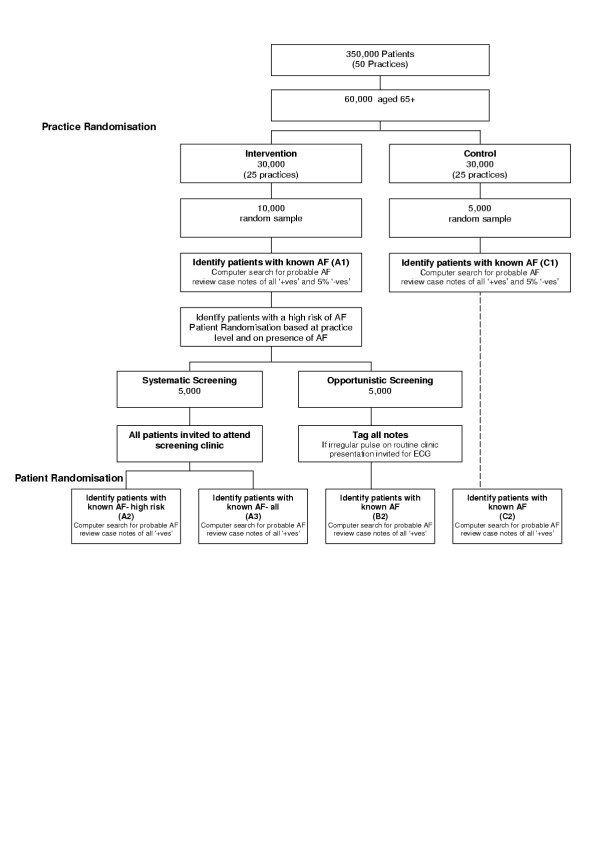
Study schema for the multi-centre randomised controlled trial.

This was a multi-centre randomised controlled trial. The study schema is shown in figure 1.

50 computerised general practices within the West Midlands were recruited through MidReC (Midlands Research Practices Consortium). This was undertaken by writing to all practices in the West Midlands and surrounding counties explaining the study and asking whether they were interested in participating. Practices showing an interest were given further information about the study and invited to attend an investigators meeting. Following the investigator meetings sixty practices interested in participating in the project were randomised (stratified based on Townsend score and practice list size): 25 as intervention, 25 as control practices with 10 reserve practices.

A computerised list of all patients aged 65+ was obtained from each practice, and from this a random sample of 10,000 patients from the intervention practices (representing approximately ^1^/_3_^rd ^of the total population of patients 65+ in this group), and 5000 from the control practices (representing approx. ^1^/_6_^th ^of the total population of patients 65+ in this group) were identified.

Patients from intervention practices were randomised, by patient, to opportunistic or systematic groups. All patients within the systematic screening arm, including those with a history of AF, were invited by post to attend a screening clinic.

For patients in the opportunistic arm, their notes were flagged within the practice to encourage practice staff to undertake pulse recording. Patients with an irregular pulse were invited to attend a screening clinic. Once this process had been undertaken, the flag was removed from the notes and returned to the research team.

The screening clinic was run by practice nurses. Patients gave informed consent. Data collected was baseline information, past medical history (including any previous diagnosis of AF), radial pulse and a 12 lead ECG. The 12 lead ECG was performed using an electronic ECG machine which allowed print-out of single lead thoracic placement ECG and a rhythm strip of lead 2 using limb leads from standard ECG.

All 12 lead ECGs were sent to two cardiologists for reporting (GL, MD). Where there was disagreement over the diagnosis a third cardiologist was used to decide. The cardiologists were asked to state whether the ECG showed AF or not, and to state whether there were any other significant abnormalities. Patients were informed of the result within two weeks. Patients with normal ECGs were informed of this, patients with any abnormality were asked to make an appointment with their GP. At the GP appointment patients with AF aged 65 – 74 were offered echocardiography. GPs were asked to make a clinical decision as to thromboprophylaxis both before and after the echocardiogram. Patients with other ECG abnormalities were managed as clinically indicated.

At the end of the screening process, GPs and Practice Nurses from both intervention practices (who had received education on ECG interpretation) and control practices (who had received no education) were sent ECGs to interpret for the presence or absence of AF. All ECGs recorded within the study were printed off as either 12 lead, single lead thoracic placement or limb lead recordings. Allocation to ECG type was random and resulted in three equal ECG groups. In order for each interpreter to read all three types of ECG, batches of ECGs were collated with the same numbers of each type of ECG. Allocation to a batch was also random. In total, there were 25 batches of ECGs to match the number of practices in each arm. The GP and Practice Nurse from the same practice read the same batch of ECGs and each batch was read by one control practice and one intervention practice. Therefore each ECG was read by two GPs and two Practice Nurses. All ECGs were anonymised, and practices did not receive any ECGs from their own practice. The interpreters were given a sheet to fill in to indicate for each ECG the presence or absence of AF. All ECGs (as 12 lead) were also analysed by the specific software package accompanying the electronic ECG and results recorded.

Patient acceptability and quality of life for different screening strategies were established using EuroQol (EQ-5D) combined together with the Speilberger 6-item Anxiety Questionnaire. EQ-5D allowed the measurement of broad aspects of quality of life. The shortened Speilberger anxiety questionnaire also has proven validity and is more specific to anxiety than is the SF-12 [17]. An adapted version of the screening-specific tool used in the Colorectal Screening Programme [18] was used to assess the acceptability of the screening process.

A random sample of 750 patients (375 screened patients and 375 opportunistically screened patients) were sent postal versions of the psychological instruments (EQ-5D and Spielberger) on entry to the study (i.e. before the intervention group has received their invitation to attend for screening). One reminder was sent a month later to non-responders. The same questionnaires were sent to the same groups plus those patients who had screened postive at the end of the screening period, approximately 17 months later. This allowed a non-randomised comparison between the effects on quality of life and anxiety in screen positive and screen negative patients.

In addition, all patients who were screened were asked to complete the acceptability and Spielberger questionnaire immediately after screening. The patient acceptability questionnaire was also administered to all patients who proceeded for echo.

The value of clinical assessment and echocardiography in risk stratification were determined in patients aged 65–74. This compared GP assessment based on the Birmingham guidelines for thromboprophylaxis in AF with any changes in recommendations for treatment once echocardiography results were available to the GP.

### Sample size and power calculations

The assumptions for the power calculations were that patients aged 65 and over represent 17% of the total population; that 40% of study population will be in the high risk group.

Also assuming that:

1. Minimum worthwhile change in detection rate was 1% for targeted screening versus routine practice. It is estimated that this change would equate to £10,000 per life year gained. This is based on the following assumptions:

a) 60% of new cases of identified AF would be suitable candidates for warfarin

b) Annual risk of stroke in this population was 5%, reduced by 60% to 2% if treated

c) Costs: £25 to screen a patient; £100 to treat with warfarin pa; £6,000 NHS costs to treat a stroke

2. 50% of patients with AF will be already known to their general practitioner (estimates range from 30% [19] to 76% [20])

3. Community prevalence of AF in this population was 6% [2]

See figure 1. It was assumed that the baseline prevalence of AF known to the practice (A1) would be 3% (i.e. half of real prevalence of 6%) and that the prevalence of known AF in the control practices would remain constant over the screening period. Thus, the change in the prevalence of known AF in the control practices between baseline to follow up (C2-C1) should be approximately 0%. The change in the GP educated arm (B2-A1) should be marginally higher and is assumed to be between 0 and 1%. The change in the systematic screening arm should, on average, be between 0 and 3% and was assumed to be approximately 2% for the total screening arm (A3-A1) and in the high risk arm (A2-A1) was approximately 3%.

All sample size calculations were for 90% power and 5% significance levels unless otherwise stated.

*a) To detect a 1% difference in detection rate between intervention (GP educated) and control practices (B2-A1) vs (C2-C1). *This requires 1,236 patients. However, since this is a difference based at the practice level of randomisation, it needed to be inflated by the design factor. Based on AF prevalence data from the EcHoES (Echocardiographic Heart of England Screening) Study [2], the between practice variance is 3.7 and the within practice variance is 246. This gave an intra-cluster correlation coefficient of 0.015. The most efficient design in this circumstance would be a cluster size of 200, which gives a design factor of 4. Therefore, 5,000 patients would be needed in 25 practices in both intervention (GP educated) and control groups.

*b) To detect a 1% difference in detection rate between intervention (Systematic screening **total** arm) and control practices (A3-A1) vs (C2-C1)*. This requires 1,236 patients but when scaled by the design factor of 4 required 5,000 patients.

*c) To detect a 1.8% difference in detection rate between intervention (Systematic screening **high risk ** arm) and control practices (A2-A1) vs (C2-C1)*. This requires 684 patients. However, since this is a difference based at the practice level of randomisation, it also needed to be inflated by the design factor. This meant that 2,736 patients would be needed in each arm. Since the ratio of patients in the two arms is 2:5 this means that 1,916 patients would be needed in the high risk arm and 4,789 in the control arm. With the 2,000 patients expected to be at high risk in this arm – resulting from the 5,000 needed for the previous comparison there were more than enough patients to detect the required difference.

Although comparison b) required fewer patients to detect the expected difference (2%) stated in the assumptions, it would be possible to detect differences as low as 1%, should the detection rate not be as high as expected.

The a), b) and c) comparisons are all at practice level randomisation.

*d) To detect a 1% difference in detection rate between high risk screening strategy and routine practice prompted by education (opportunistic arm) (A2-A1) vs (B2-A1).*This requires 1,236 patients in both the high risk systematic screening and the GP educated (opportunistic) screening arms of the intervention practices assuming the high risk screening detects a 1% increase and opportunistic screening detects 0% increase. Should the increased detection rates be higher in each arm (1.7% in the high risk arm and 0.7% in the opportunistic arm) then this could require 2,686 patients in each arm. However, since there is a ratio of 2:5 patients in these arms there will be sufficient patients as only 1,880 are needed in the high risk arm and 4,700 in the opportunistic arm to be able to detect this 1% difference*.*

e) To detect a 1% difference in detection rate between total screening strategy and routine practice prompted by education (opportunistic arm) (A3-A1) vs (B2-A1).This requires 3,300 patients in both the total screening and the GP educated (opportunistic) screening arms of the intervention practices.

*f) To detect a relative risk (RR) of 2 (1% detection rate difference) between total population and high risk screening (A3 vs A2).*It was assumed that 40% of the study population fall into the high risk group, and the prevalence of undetected AF is 3%. This meant that 1,434 patients would be needed in each of the two risk groups to detect a two fold difference in risk (i.e. RR of AF in high risk as compared to low risk group is 2). This RR of 2 equates to an increase in AF detection rate from 3% in the total population arm to 4% in the high risk arm. Since there was a 40:60 split in the two risk groups unequal sample size calculations only require a minimum of 1,200 patients in the high risk group and 1,800 in the moderate/low risk group. This was achievable with a screening arm of 5,000 patients, as there would actually be 1,320 in the high risk group and 1,980 in the moderate risk group if a 66% screening acceptance rate was assumed.

### Sample size for quality of life assessment

Although some of the variances are from North American populations we have no reason to suspect that the variation will be different in a British population since data from the ECHOES study on the SF36 gives variations very similar to the North American norms.

### Spielberger

The shortened (6-item) version of the Spielberger state anxiety questionnaire has been validated and used in populations different from that under consideration in SAFE, namely it tends to have been used in young and mostly female populations [17,21,22]. The variance obtained from these papers appears to be approximately 144 for Marteau [17] but higher for the Ubhi [22] paper. However, the women in the latter paper were being informed of major illness outcomes (either benign or malignant breast cancer). A full Spielberger on people undergoing physiological tests also gave a variance of the order of 144 [23]. The full version of the Spielberger state anxiety when used with an elderly population also seems to give a variance that is not too far from the previously mentioned papers being 188.8 [23]. Taking this latter value as being the nearest to our population we can detect a 4 point difference in the mean values obtained with 249 patients in each arm.

### EQ5D

#### The VAS scale

The VAS variance as reported for an elderly population aged 75 and over was 365 [24] but for a group of recovered stroke patients (ages not given) it was approximately 100 [25]. Taking the former value as a worst case this means it will be possible to detect a 6% difference between groups on the VAS with 213 patients within each group.

#### The Utility index

This was reported in different ways in the Johnson [24] and Dorman [25] papers. Using the utility values from the Dorman et al paper the variance is approximately 0.066 and this allows us to detect a 0.1 difference with 139. Using the Johnson paper the variance is approximately 576 and using this means that we can detect a mean change of 7 with 247 patients in each arm.

### Statistical analysis

Intention to treat analysis will be used.

Any previously known Atrial Fibrillation cases will be subtracted from the totals obtained at the end of the study to ensure there is no double counting in the incidence figures.

Chi-squared, independent t-tests and log-linear models will be used to describe demographic data. If there are differences between the groups this may need to be adjusted for in later analyses.

### Primary objective: to determine baseline prevalence and incidence of AF on a variety of screening strategies

Proportions and rates will be used as the measures of prevalence and incidence.

The independent t-test and ANOVA with random effects (as appropriate) will be used to examine the detection rate differences between the intervention and control screening strategies. Should the data be strongly non-normal a non-parametric equivalent will be used.

### Secondary objectives

a) to assess patient acceptability and impact on QoL of different screening strategies

The independent and related t-test and ANOVA will be used to examine the differences between the intervention screening strategies on the Spielberger and EuroQol EQ5D. Should the data be strongly non-normal a non-parametric equivalent will be used. Chi squared tests will be used on the screening tool.

b) to assess the value of echocardiography in risk stratification for thromboembolic disease in patients with AF

McNemar's test will be used to see whether there is any significant change in the doctor opinion on risk of CVA and treatment decision before and after echo screening.

c) to evaluate the most cost effective method of test interpretation

The cost effectiveness will be covered in the economic section. However, the use of sensitivity, specificity, Cohen's κ and conditional logistic modelling will allow for comparison of the various methods for detecting AF between the GPs, nurses and consultants.

d) to evaluate the most cost effective method of screening

This will be covered in the economic analysis section.

Multivariate and logistic modelling analyses will be undertaken in order to determine which markers might be the best predictors of the presence of AF. This will act as confirmatory analysis for the risk factors used in the screening strategy to define a high risk patient.

### Economic analysis

#### Framework for the economic analysis

This trial evaluated a large number of alternative screening scenarios for identifying atrial fibrillation (2 screening strategies i.e. target v population; 6 screening methods i.e. pulse plus 3 types of ECG if pulse abnormal, and 3 types of ECG regardless of pulse; and 4 screening test interpretations, making 2*6*4 = 48 plus control and opportunistic screening = 50). The study has been powered to detect a difference in the targeted versus population arms, and in systematic screening versus routine clinical practice. However, the economic analysis will compare the cost-effectiveness of all alternative screening approaches using a modelling framework, whereby data will be drawn from the trial (where appropriate and available) and from external sources.

The use of a modelling approach allows the timescale for the economic analysis to be extended beyond the follow-up period allowed for in the trial. The use of such extrapolation will enable estimation of the incremental cost-effectiveness ratios (ICERs) for each approach:

Incremental cost per case detected

Incremental cost per life year gained

Incremental cost per quality-adjusted life year (QALY) gained

#### Data on consequences

The use of EQ-5D allows the measurement of broad aspects of quality of life. EQ-5D allows changes in health status to be measured but also valued, using the University of York Measurement & Valuation of Health general population survey tariff [27].

#### Cost data

The cost analysis adopted a broad perspective to include costs incurred within the health sector and by patients and carers. Data collection was undertaken on all trial patients in order to allow a stochastic cost analysis to be conducted. The focus of the data collection will be upon the key cost drivers which will include:

a) the resources required to establish screening (invitation to patient, follow ups, communication of results),

b) the time taken to carry out the various tests, and

c) the cost of the equipment (expressed as cost per test).

a), b) and c) will be based on data collected in the study.

The analysis adopted an incremental approach such that data collection concentrated on resource use differences between alternative screening scenarios. The process of collecting data on resource use was undertaken separately from data collection on unit costs. Resource use data on the screening process was principally collected within the trial. Unit costs were collected from published sources and a representative sample of NHS providers in order to increase generalisability. The methods used in collecting data will include patient questionnaires (see above) and review of patient records (both GP and hospital). Data on private costs were collected from a survey of a sub-cohort of the trial population.

#### Cost effectiveness analysis

The plan for the analysis is:

1. Report a cost consequence analysis, which will involve providing a full description of all important results relating to costs and consequences.

2. Conduct both a cost-effectiveness analysis and a cost utility analysis (using data on true positive cases detected as the measure of effect and data on EQ-5D to estimate QALYs).

An incremental approach will be used in order to compare the large number of alternative screening strategies.

We are interested in comparing the mean costs per patient since our concern is with predicting overall programme costs. However, the data on costs are likely to have a skewed distribution. Therefore, the plan for the analysis of costs is:

1. To explore the nature of the distribution of costs

2. If required, to use non-parametric comparison of means (e.g. bootstrapping)

3. If the distribution of the data is approximately normal, parametric methods will be used.

This approach is in line with recent recommendations [26].

If missing data are a problem at the economic analysis stage, then imputation techniques will be employed.

Longer term costs and consequences will be explored by extrapolating beyond the end of the trial using a modelling framework using data from a range of trial and non-trial sources. The precise form of modelling is yet to be determined, but is likely to be either Markov or Discrete Event Simulation, depending upon the extent to which the Markov assumptions are justified. An advantage of using such an approach is that it will allow the additional costs of increasing survival to be explicitly incorporated into the analysis. In particular, modelling will provide estimates of the optimal frequency of screening for AF, based on the estimates of incidence and prevalence from the trial.

Identifying untreated patients with AF will have implications for service provision. These will depend upon the prevalence of atrial fibrillation, the screening mechanism employed, the use made of echocardiography, and the additional requirements for anticoagulation monitoring. The separate parallel study, BAFTA, will provide empirical data that will allow such implications for service provision to be assessed. The modelling exercise, which will draw on both SAFE and later BAFTA results, will combine best estimates of both screening and anticoagulation options.

The robustness of the results of the economic analysis will be explored using sensitivity analysis [27]. This will explore uncertainties in the trial based data itself, the methods employed to analyse the data and the generalisability of the results to other settings. Uncertainty in the confidence to be placed on the results of the economic analysis will be explored by estimating cost-effectiveness acceptability curves. These plot the probability that the intervention is cost-effective against threshold values for cost-effectiveness.

### Inclusion and exclusion criteria

#### Inclusion criteria

Patients aged 65 years or over (65+).

#### Exclusion criteria

Patients who were terminally ill.

#### Randomisation

Randomisation of practices and patients was performed by statisticians from the Department of Primary Care and General Practice at The University of Birmingham. Cluster randomisation of practices to intervention or control was stratified by Townsend quartiles and practice size. Computer searches were carried out to identify cases of known AF, within the sample of patients identified above, using a published strategy [15]. The randomisation of patients within the intervention practices ensured that the study patients in each practice were divided equally between systematic and opportunistic screening arms and also that there was an even distribution of patients with known AF between the two arms. Patients within the systematic screening arm were identified by computerised record searching as either being at high risk (target population) or moderate risk (non-target population) of AF by recognised criteria [28,11].

#### Cleaning of lists post randomisation

Following initial sampling of the total population the list of patients from each practice were returned to the practices who were asked to remove any patients who had died, moved or were terminally ill. Patients removed following this process were replaced with patients from a reserve list, which had been randomised at the same time as those on the initial lists.

#### Patient information and consent

Patients aged 65+ who were selected to the systematic screening arm received an information sheet with an invitation letter to attend the ECG clinic. Entry to the trial was discussed with the practice nurse at the clinic. The practice nurse then obtained written consent from those patients who were willing to participate.

Study patients found opportunistically to have an irregular pulse were given an information sheet and invited to attend the screening clinic.

#### Practice staff education and ECG training

GPs and other members of the primary health care team in the intervention practices attended investigator days at which they were given educational materials informing them of the importance of detection of AF, and the treatment options that are available. The materials encouraged them to consider opportunistic screening of patients.

Members of the primary health care team in control practices received no educational input from the research staff.

Practice nurses attended an ECG training day prior to starting the ECG screening clinics. Training included how to perform an ECG (using the Biolog) to ensure a standardised high quality tracing and basic ECG interpretation (specifically how to identify AF).

### Computerised and note searches of GP records

#### Prevalence and incidence data

Computer searches were carried out to identify cases of probable AF in the 15,000 study patients using a published strategy [15]. Searches were tailored towards the information that is held on computer in each practice. If practices hold AF registers, or use READ diagnosis coding, then these were used. In addition, a search was carried out to identify prescriptions of digoxin, a beta-blocker, a class 1,3 or 4 anti-arrhythmic agent, aspirin or warfarin. This information was recorded into computerised case report forms.

Case notes of patients identified as 'known' or 'probable' AF in any of these computer searches were reviewed for mention of a diagnosis of AF. AF diagnosis were drawn from hospital letters stating the existence of the condition or ECG recordings from the last 5 years.

An additional 5% random sample of case notes of patients not identified as 'known' or 'probable' AF by computer searching were reviewed (750 in all) to estimate how many other patients who are known to have AF were not identified by the computer searches. If this had revealed a significant number of extra cases of known AF, then the sample size for manual searching would have been increased to allow a precise estimate of the baseline rate of known AF. Unidentified extra AF cases were not found to be significant so no additional note search was required.

The same computer searches on both intervention and control practice patients notes were performed prior to, and 12 months after, commencement of screening.

#### Screening clinics

All patients in the systematic screening arm and those found to have an irregular pulse in the opportunistic screening arm of the study were invited to attend an ECG screening clinic. At the clinic the practice nurse explained the aims of the study and answered any questions about the study. Written informed consent to participation in the study was obtained from the patient. The nurse then recorded baseline information on age, sex, present smoking and alcohol status and past medical history, including previous diagnosis of AF, and any treatment the patient may be receiving for AF. Radial pulse rate, and whether regular or irregular, was noted. A 12 lead ECG, the gold standard by which other traces were compared, was then recorded using the Biolog machine, which was also able to produce a trace corresponding to the single lead thoracic placement and a rhythm strip of lead II. Finally, the patient was asked to complete an acceptability questionnaire.

## Discussion

This study will identify the most cost-effective strategy for identifying atrial fibrillation in patients aged 65 and over. The policy implications will be dependent on the findings and one of the strengths of the current study is the utilisation of modelling techniques to investigate the implications of different screening strategies and frequency of screening within different health care environments. The initial draft of the report has been submitted to the Department of Health and publication of the results should be expected later this year (2004).

## Competing interests

None declared.

## Authors' contributions

RH and DF were principal investigators. EM and SJ were project managers. DS collected data and prepared the first draft of the paper. JM, EM, JR, SB, MD and GL all contributed to the project design. All authors read and approved the final manuscript.

## Pre-publication history

The pre-publication history for this paper can be accessed here:


